# Abstracts

**DOI:** 10.1155/S1463924686000111

**Published:** 1986


Journal of Automatic Chemistry/*Journal of Clinical Laboratory Automation*, Vol. 8, No. 2 (April–June 1986), pp. 45–48

### Abstracts

### Page 49

### Computer acquisition and analysis of data in enzymatic-fluorimetric-continuous-flow methods for the measurement of glucose, lactate, pyruvate, alanine, glycerol and 3-hydroxybutyrate in human blood

C. S. Hetherington *et al.*

A program is described to acquire and process data using an Apple computer in the determination of human blood glucose, lactate, pyruvate, alanine, glycerol and 3-hydroxybutyrate. The assay methods are enzymatic and combine continuous-flow systems with fluorimetry. Particular attention in the program is given to the necessity to correct for the blank fluorescence present in perchloric extracts of blood, although it can also be used for a variety of assays where 'blank' runs are not required. Using peak height measurements the blood metabolite concentration is determined by a software package consisting of four subprograms, which allow flexibility, although a rigid sequence of recurring groups of standards, controls and samples is necessary. In the 'acquisition' subprogram, data can be acquired from three fluorimeters simultaneously. The 'amend' subprogram is used to modify data by addition or deletion. With the 'analysis' subprogram, the metabolite concentration in the perchloric acid extracts is determined using either the means of all the groups of standards or with separate groups of standards. In the 'collate' subprogram all the information concerning the samples, including dilution factors, patient identification and controls, is collated and the results printed in a coherent format. The correlation between the computer determined results and those determined by conventional reading of chart recorder traces is excellent. Considerably less time is required for processing the computer data.

*French and German summaries will be printed in our next issue.*

### Page 56

### Flow Injection Analysis: A new tool to automatize extraction processes

M. D. Luque de Castro

An overview of a Flow Injection Analysis (FIA) liquid-liquid extraction combination is presented. Its most significant features, its theory, basic components, applications and advantages over other automatic methods incorporating continuous extraction are also shown.

*French and German summaries will be printed in our next issue.*

### Page 63

### An evaluation of the Coulter DACOS analyser

A. E. Hurrell, R. A. Hall and J. L. Robinson

An evaluation is presented of the DACOS (Discrete Analyser with Continuous Optical Scanning), which has been developed commercially by Coulter Electronics Ltd, Northwell Drive, Luton, Bedfordshire LU3 3RH, UK and Coulter Electronics, Inc., Hialeh, Florida, USA.

The instrument is a high-capacity discretionary analyser, combining the advantages of centrifugal analysis and sequential continuous operation. Its main feature is that the optical system itself rotates continuously around the samples. It uses semi-disposable cuvettes which are automatically laundered. Instrument control, reaction monitoring and data processing, together with test-programming, are available through a dedicated microcomputer.

A description is given of the instrument and its operation, including the types of assay that can be performed and their calibration.

The evaluation was carried out over three months using a variety of methods, and the findings are discussed. Accuracy, precision and linearity were satisfactory. Staff found the analyser easy to use and maintenance was simple. Emergency analyses could be accommodated without difficulty and the small sample volumes required were ideally suited to paediatric work.

One or two minor safety problems were identified. Noise and sample-to-sample interaction were initially considered unsatisfactory, but were later improved. The major problem found was unreliability, which should be resolved as software revisions are made by the manufacturer.

Given a test: sample ratio of 2 : 1, the instrument can process up to 1000 tests per day. This would cope with the work-load of a small- or medium-sized district general hospital. With the addition of ion-selective electrodes for sodium and potassium estimation, and the provision of bi-directional interfacing, the DACOS could seriously be considered as a replacement for traditional profiling techniques.

### Evaluation d'un analyseur Dacos Coulter

A. E. Hurrell, R. A. Hall and J. L. Robinson

Une évaluation du système DACOS (Discrete Analyser with Continuous Optical Scanning) qui a été développé par Coulter Electronics Ltd, Northwell Drive, Luton, Bedfordshire, LU3 3RH, UK et Coulter Electronics, Inc., Hialeh, Florida, USA, est présentée.

L'instrument est un analyseur discret à haute capacité combinant les avantages de l'analyse centrifugale et l'opération séquentielle en continu. Sa propriété principale est que le système optique tourne en continu autour des échantillons. Le système utilise des cuvettes qui sont automatiquement nettoyées. Le contrôle de l'instrument, de la réaction et du traitement de données ainsi que les programmes de test sont exécutés à partir d'un microordinateur dédié.

Une description de l'instrument et de son mode opératoire est donnée incluant le genre d'analyses qui peuvent être exécutées et leur calibrage.

L'évaluation a été faite pendant une période de trois mois utilisant une variété de méthodes et les résultats sont discutés. L'exactitude, la précision et la linéarité étaient satisfaisantes. Les employés ont trouvé que l'analyseur était facile à utiliser et l'entretien était simple. Les analyses urgentes ont été faites également sans difficultés et les petits volumes d'échantillons requis se prêtent idéalement au travail pédiatrique.

Un ou deux problèmes de sécurité mineure ont été identifiés. Le bruit de fond et l'interaction entre échantillons étaient considérés insatisfaisants au début, mais ont été améliorés au cours de l'étude. Le seul problème majeur était un manque de fiabilité, un problème qui devrait être résolu aussitôt que les révisions de logiciel seront faites par le producteur.

L'instrument peut effectuer jusqu'à 1000 analyses par jour étant donné une relation analyses/échantillon de 2 à 1. Ceci correspond environ à la demande d'un hôpital général de district petit ou moyen. Avec l'addition d'électrodes ions-sélectives pour le dosage du sodium et du potassium et la possibilité d'interface bidirectionnelle, le DACOS peut sérieusement être considéré comme remplacement pour les techniques traditionnelles.

**Evaluation des Coulter Dacos Analysengerätes**

A. E. Hurrell, R. A. Hall and J. L. Robinson

Die Arbeit evaluiert DACOS (Batch-Analysengerät mit kontinuierlicher optischer Abtastung), der von Coulter Electronics für kommerzielle Zwecke entwickelt wurde.

Das Gerät ist ein Hochleistungs-Analyseninstrument, das die Vorteile des Zentrifugenprinzips mit dem des sequentiellen kontinuierlichen Betriebs vereint. Sein Hauptmerkmal ist das optische System selbst, das kontinuierlich um die Proben herum rotiert. Es benützt halb-weg-werfbare Küvetten, welche automatisch gewaschen werden. Gerätesteuerung, Reaktionsverfolgung und Datenverarbeitung sowie die Testprogrammierung werden von einem nur dafür eingesetzten Mikrocomputer besorgt. Eine Beschreibung des Gerätes und seines Betriebs umfasst auch die Arten der Analysen und deren Eichung, die durchgeführt werden können.

Die Evaluation wurde während dreier Monate, unter Benützung einer Reihe von Methoden durchgeführt, und die Ergebnisse wurden besprochen. Genauigkeit, Präzision und Linearität sind zufriedenstellend. Das Betriebspersonal erachtete des Gerät als leicht bedienbar, und die Wartung ist einfach. Eilanalysen konnten ohne Schwierigkeiten berücksichtigt werden. Die erforderlichen kleinen Probenvolumina eigneten sich ideal für paediatrische Arbeiten.

Ein oder zwei kleinere Sicherheitsprobleme wurden identifiziert. Signalrauschen und Wechselwirkung zwischen Proben waren anfänglich ungenügend aber wurden später verbessert. Ein grösseres Problem stellte die ungenügende Zuverlässigkeit dar, die jedoch durch Software-Ueberarbeitungen des Herstellers beseitigt werden sollen.

Bei einem Verhältnis von Analysen zu Proben von 2:1 kann das Gerät bis zu 1000 Analysen pro Tag durchführen. Damit kann die Arbeitslast eines kleinen bis mittleren Regionalspitals bewältigt werden. Durch Zufügen von ionen-selektiven Elektroden für die Bestimmung von Natrium und Kalium und die Einrichtung eines bidirektionalen Interface kann der DACOS ernsthaft für den Ersatz traditioneller Profiltechniken in Erwägung gezogen werden.

**Page 70**

**Automatation of a flow-injection system for multispeciation**

Juan Ruz, Antonia Torres, Angel Ríos, M. D. Luque de Castro and Miguel Valcárcel

An automatic flow-injection system, which is based on the integration of two detectors (photometric and potentiometric), is presented. The system allows absorbance-pH data to be collected from every sample by means of an on-line microprocessor, which, with a suitable calculation program, provides the concentration of up to nine species of chromium present in the sample. A 'reversed' configuration and the asymmetric merging zones mode are used to discriminate between the different oxidation states of chromium.

*French and German summaries will be printed in our next issue.*

**Page 75**

**Evaluation of derivative spectra for the selective determination of drugs: quantitation of theophylline with phenobarbital and light-scattering components**

P. B. Arnoudse and H. L. Pardue

This paper describes the evaluation of zeroth-, first-, and second-derivative spectra for the quantitation of drugs in mixtures. In particular, single-wavelength and multi-wavelength data-processing methods are compared for the quantitation of theophylline in the presence of phenobarbital and a light-scattering component. For 0–25 mg/l of theophylline in the presence of variable amounts of phenobarbital and the light-scattering component, single- and multiwavelength second-derivative data yielded least squares equations of computed ($y$) versus prepared ($x$) concentrations of $y = 1{\cdot}00 \times + 0.52$ mg/l and $y = 0{\cdot}98 \times + 0{\cdot}04$ mg/l, respectively, with standard errors of estimate of 0·32 and 0·04 mg/l. First-derivative data yield slightly larger intercepts and standard errors and absorption data yield much larger intercepts and standard errors.

**Evaluation de spèctres dérivés pour la détermination sélective de médicaments: quantification de la théophiline contenant du phénobarbital et des composantes dispersant la lumière**

P. B. Arnoudse and H. L. Pardue

Cette publication décrit l'évaluation de spèctres ainsi que de leurs premières et secondes dérivées pour quantifier des médicaments dans des mixtures. En particulier, des méthodes de traitement de données pour une longeur d'ondes fixe et multiples-longueurs d'ondes sont comparées pour la quantification de la théophiline en présence du phénobarbital et d'une composante dispersant la lumière. Pour 0–25 mg/l de théophiline en présence de quantités variables de phénobarbital et de la composante dispersant la lumière, les secondes dérivatives pour longueur d'ondes simple et multiple, donnaient des équations de moindre carré pour le signal en fonction de la concentration de y = 1·00 × + 0·52 mg/l et de y = 0·98 × + 0·04 mg/l respectivement avec des erreurs standard pour la valeur estimée de 0·32 et 0.04 mg/l. Les données basées sur la première dérivée montraient des valeurs d'interception et des erreurs standard légèrement plus élevées et les mesures d'absorption donnaient des interceptions et des erreurs standard beaucoup plus élevées.

**Auswertung von Ableitungsspektren für die selektive Bestimmung von Drogen: Quantisierung von Theophyllin in Anwesenheit von Phenobarbital und lichtstreuenden Komponenten**
P. B. Arnoudse and H. L. Pardue

Diese Arbeit beschreibt die Auswertung der nullten, ersten und zweiten Ableitung von Spektren für die Quantisierung von Drogen in Gemischen. Im besonderen werden Einwellenlängen- und Multiwellenlängenmethoden der Datenverarbeitung für die Quantisierung von Theophyllin in Anwesenheit von Phenobarbital und lichtstreuenden Komponenten verglichen. Für 0–25 mg/l Theophyllin in Anwesenheit von verschiedenen Mengen von Phenobarbital ergaben die Einwellenlängen- und Multiwellenlängendaten der zweiten Ableitung die Regressionsgeraden y = 1·00 × + 0·52 mg/l und y = 0·98 × + 0·04 mg/l für berechnetes y gegen hergestellte Konzentrationen X, mit Standardabweichungen von 0·32 bzw. 0·04 mg/l. Die Daten der ersten Ableitung ergeben leicht grössere Achsenschnitte und Standardabweichungen. Die Absorbtionsdaten dagegen ergaben wesentlich grössere Achsenschnitte und Standardabweichungen.

---

**Page 80**

**Evaluation of fluorescence excitation transfer immunoassay for the measurement of plasma cortisol**
J. Calvin *et al.*

Fluorescence excitation transfer immunoassay is a suitable technique for the measurement of plasma cortisol. The reagents, once reconstituted, are stable for at least three months. The method shows no interference from bilirubin or lipaemia up to high levels. Haemolysed specimens give falsely raised results and prednisolone causes significant positive interference. The precision of the assay compares favourably with the radioimmunoassay used routinely. The assay is simple to perform and reliable. Comparison with an HPLC technique indicated a significant bias; reflecting the positive bias seen with Advance results in external quality assessment materials when compared with GCMS target values. Comparison with RIA yielded a wide scatter, although the regression parameters gave a slope of 0·97. The discrepancies are probably due to differing specificities of the antibodies employed.

**Evaluation de l'immunoassay par transfert d'excitation de fluorescence pour le dosage du cortisol de plasma**
J. Calvin *et al.*

L'immunoassay de transfert d'excitation de fluorescence est une technique convenant pour le dosage du cortisol dans le plasma. Les réactifs reconstitués sont stables pour au moins 3 mois. La méthode montre aucune interférence due à la bilirubine ou à la lipémie même à niveaux élevés. Des échantillons hémolisés donnent des résultats trop élevés et la prednisolone cause une interférence positive significative. La précision de cette méthode de dosage se compare favorablement avec le radioimmunoassay utilisé en routine. La méthode est simple et sûre. La comparaison avec la technique de HPLC indique une déviation significative reflettant une déviation positive observée lors du contrôle de qualité externe pour des matériaux comparés avec les valeurs GCMS. La comparaison avec le radioimmunoassay montre une grande dispersion bien que les paramètres de régression donnent une pente de 0·97. Les discrépances sont probablement dues aux spécificités différentes des anticorps employés.

**Auswertung der Fluoreszenz-Anregungsübertragung-Immunomethode für die Bestimmung des Plasmacortisol**

J. Calvin *et al.*

Die Fluoreszenz-Anregungsübertragung-Immunomethode ist eine geeignete Technik für die Bestimmung von Plasma-Cortisol. Die Reagenzien, einmal hergestellt, sind für mindestens drei Monate stabil. Die Methode zeigt keine Interferenz mit Bilirubin oder Lipaemia, auch bei hohem Spiegel. Haemolysierte Proben geben fälschliche überhöhte Werte, und Prednisolon verursacht signifikante positive Interferenz. Die Präzision der Methode vergleicht sich günstig mit der Routine-Radioimmunomethode. Der Test ist zuverlässig und einfach durchführbar. Vergleiche mit einer HPLC-Methode ergaben eine signifikante systematische Abweichung. Ein Vergleich mit RIA ergab eine grosse Streuung, obwohl die Regression eine Steigung von 0·97 ergab. Die Diskrepanzen wurden wahrscheinlich durch unterschiedliche Spezifitäten der verwendeten Antikörper verursacht.

**Page 85**

### The influences of the between- and within-run components of variation on the mean rule

P. Douville *et al.*

Previous work with a specific error model has indicated that there is degradation in the performance characteristics of the mean rule when it is used for the quality control of analyses with significant between-run variation. The selection of the error model can markedly affect the performance characteristics of the mean rule. An alternate error model for the mean rule is proposed, which more realistically simulates its application in the clinical laboratory. This model indicates that the mean rule's probabilities of error detection and false rejection are not compromised by significant between-run components of variation. The use of the mean rule is recommended for the quality control of analytical methods using multiple controls regardless of the magnitude of the between-run variation.

### L'influence des composantes de variation entre et intra-série sur la règle de la moyenne

P. Douville *et al.*

Un travail antérieur avec un modèle d'erreur spécifique nous a indiqué qu'il y a dégradation des caractéristiques de performance de la règle de la moyenne quand elle est utilisée pour le contrôle de qualité d'analyses avec variations significatives entre séries. Nous avons trouvé que la sélection du modèle d'erreurs peut affecter les caractéristiques de performance de la règle de la moyenne. Nous proposons un modèle d'erreur alternatif pour la règle de la moyenne qui simule de façon plus réaliste son application dans le laboratoire clinique. Ce modèle indique que la probabilité de détection d'erreurs de la règle de la moyenne et d'une réjection fausse ne sont pas compromises par les composantes de variation significatives entre séries. Nous recommandons l'utilisation de la règle de la moyenne pour le contrôle de qualité de méthodes analytiques utilisant des contrôles multiples indépendamment de la grandeur des variations entre séries.

### Einflüsse der Variationskomponenten innerhalb und von Serie zu Serie auf die Mittelwerts-Regel

P. Douville *et al.*

Frühere Arbeiten mit einem spezifischen Fehlermodell deuteten an, dass eine Beeinträchtigung der Leistungsfähigkeit der Mittelwerts-Regel eintritt, wenn sie für die Qualitäskontrolle von Analysen eingesetzt wird, die von Serie zu Serie signifikante Variationen zeigen. Wir stellten fest, dass die Wahl der Fehlermodelle deutlich die Leistung der Mittelwerts-Regel beeinflusst. Wir schlagen ein anderes Fehlermodell für die Mittelwerts-Regel vor, das realistischer die Anwendung im klinischen Labor simuliert. Dieses Modell zeigt, dass die Wahrscheinlichkeiten der Fehlerdetektion und der fälschlichen Rückweisung durch die Mittelwerts-Regel nicht durch signifikante Variationskomponenten von Serie zu Serie nicht kompromitiert werden. Wir empfehlen die Mittelwerts-Regel für die Qualitätskontrolle von Analysenmethoden unter Verwendung von vielfachen Kontrollen unabhängig von der Grösse der Variationen von Serie zu Serie.

### ANALYTICON 86

Analyticon 86, to be held at the Commonwealth Institute in London 23 to 25 September 1986, is being upgraded from a 10-session meeting to include a total of 18 sessions plus poster displays. More intensive coverage of more topics of direct interest to practising laboratory personnel will be included in this three-day multidisciplinary meeting. Speakers are being invited from Europe and the USA to present the latest advances and concepts in laboratory technology.

A special seminar session on laboratory robotics will be presented by Dr Grover Owens of the Proctor and Gamble Company, USA. The emphasis in this session will be on users' experiences and particularly with small-scale systems.

Another topic area of sufficient importance to warrant two sessions in the Analyticon 86 programme is spectroscopy. One session will be on multi-component spectroscopy, the other on atomic spectroscopy. Another special seminar will be presented by Dr Bernard Vandeginste from the University of Nijmegen in The Netherlands on 'the analysis of mixtures—chemometrics for data analysis in liquid chromatography with diode array detectors and for multicomponent analysis in diode array spectrometers'.

There will also be specific sessions on chromatography within the programme. It is intended that these will deal in further depth with diode array detection and with biochemical applications of HPLC.

*Further information from Scientific Symposia Ltd, 33–35 Bowling Green Lane, London EC1R 0DA. Tel.: 01 837 1212.*

